# Adsorptive Removal Behavior of Two Activated Carbons for Bis(2-ethylhexyl) Phosphate Dissolved in Water

**DOI:** 10.3390/toxics13080624

**Published:** 2025-07-25

**Authors:** Lifeng Chen, Jing Tang, Zhuo Wang, Hongling Wang, Wannian Feng, Junjie Chen, Qingqing Yan, Shunyan Ning, Wenlong Li, Yuezhou Wei, Di Wu

**Affiliations:** 1 School of Nuclear Science and Technology, University of South China, 28 Changsheng West Road, Hengyang 421001, China; chenlf@usc.edu.cn (L.C.); tangj@stu.usc.edu.cn (J.T.); wangz14@foxmail.com (Z.W.); fengwn@stu.usc.edu.cn (W.F.); chenjj-2004@foxmail.com (J.C.); yanqq2024@163.com (Q.Y.); ningshunyan@usc.edu.cn (S.N.); liwenlong@usc.edu.cn (W.L.); yzwei@usc.edu.cn (Y.W.); 2 Key Laboratory of Advanced Nuclear Energy Design and Safety, Ministry of Education, 28 Changsheng West Road, Hengyang 421001, China; 3 Institute of Resources Utilization and Rare Earth Development, Guangdong Academy of Sciences, 363 Changxing Road, Guangzhou 510650, China; wanghongling@grre.gd.cn; 4 School of Nuclear Science and Engineering, Shanghai Jiao Tong University, 800 Dong Chuan Road, Shanghai 200240, China

**Keywords:** bis(2-ethylhexyl) phosphate, activated carbon, adsorption, removal, acetone

## Abstract

Bis(2-ethylhexyl) phosphate (P204) is widely used in extraction processes in the nuclear and rare earth industries. However, its high solubility in water results in high levels of total organic carbon and phosphorus in aqueous environments, and may also lead to radioactive contamination when it is used to combine with radionuclides. In this paper, we characterized a coconut shell activated carbon (CSAC) and a coal-based activated carbon (CBAC) for the adsorption of P204 and then evaluated their adsorption performance through batch and column experiments. The results found that, except for the main carbon matrix, CSAC and CBAC carried rich oxygen-containing functional groups and a small amount of inorganic substances. Both adsorbents had porous structures with pore diameters less than 4 nm. CSAC and CBAC showed good removal performance for P204 under low pH conditions, with removal efficiencies significantly higher than those of commonly used adsorption resins (XAD-4 and IRA900). The adsorption kinetics of P204 conformed to the pseudo-second-order kinetic model, and the adsorption isotherms conformed to the Langmuir model, indicating a monolayer chemical reaction mechanism. Both adsorbents exhibited strong anti-interference capabilities; their adsorption performance for P204 did not change greatly with the ambient temperature or the concentrations of common interfering ions. Column experiments demonstrated that CSAC could effectively fix dissolved P204 with a removal efficiency exceeding 90%. The fixed P204 could be desorbed with acetone. The findings provide an effective method for the recovery of P204 and the regeneration of spent activated carbon, which shows promise for practical applications in the future.

## 1. Introduction

Organic phosphoric acid extractants are widely used in the nuclear and rare earth industries due to their good extraction performance [1,2,3,4,5]. Bis(2-ethylhexyl) phosphate (P204), a representative organic phosphoric acid extractant, is a colorless to yellowish transparent viscous liquid containing one phosphoric acid group (–PO(OH)_2_) and two 2-ethylhexyl branches. The extractant dissolves, distributes, and dissociates in two phases; thus, a part of the extractant is often dissolved in the aqueous phase during extraction processes, resulting in increased phosphorus and total organic carbon (TOC) contents in water. Phosphorus and TOC are harmful to the human body, and their contents are important indicators for evaluating water quality. Due to its high solubility in water, the content of P204 in aqueous media can reach several hundred ppm [6]. Furthermore, once combined with radionuclides, soluble P204 may also result in radioactive environmental pollution. Therefore, efficient removal methods for P204 dissolved in water are needed.

To date, reports on the removal of dissolved P204 from aqueous solutions remain scarce. Because P204 is an organophosphorus extractant, the treatment of wastewater polluted with other organophosphorus or organic compounds can be used as a reference. The treatment methods for wastewaters containing organophosphorus compounds mainly include biodegradation [7,8,9,10], electrochemical degradation [11,12], chemical degradation [13,14], radiation degradation [15], membrane filtration [16,17], liquid–liquid extraction [18,19], Fenton oxidation [20,21,22], and solid-phase adsorption [23,24,25,26,27,28,29,30]. Among these, solid-phase adsorption is widely used in organic wastewater treatment due to its simplicity, high efficiency, and recyclable adsorbate. Reported adsorbents can generally be classified into porous polymers [29,31,32,33], activated carbons (ACs) [24,25,34], and metal–organic frameworks (MOFs) [16,23,35], etc. For example, Pandey et al. [29] studied the adsorption behavior of XAD-4 resin for dissolved tri-n-butyl phosphate (TBP) in water as early as 1997. They found that columns filled with XAD-4 resin effectively fixed TBP in water under dynamic conditions, with a removal rate close to 100%. Zhuang et al. [28] hydrothermally synthesized UiO-66-NH_2_, an MOF with a defect structure, for the removal of diclofenac in solution. The maximum adsorption capacity of UiO-66-NH_2_ reached 555 mg/g, and the adsorbent showed relatively fast adsorption kinetics. Yin et al. [36] reported a red mud modified with polypyrrole that achieved equilibrium adsorption capacities of 32.9 and 54.7 mg/g for inorganic and organic phosphorus in solution, respectively, along with a wide pH range. Bakhiia et al. [25] studied activated pinecones, graphene oxide, and AC for the removal of dissolved TBP in water. The TBP adsorption capacity of the carbon materials increased with increasing specific surface area, and adsorbents could be regenerated by thermal treatment, followed by washing with water. Although the above-mentioned adsorption materials are often used to remove organic materials from wastewater, they are not always effective. Their performance strongly depends on multiple factors, including the hydrophilicity or hydrophobicity of the organic molecules, molecular size, acidity, temperature, and presence of coexisting ions. P204, an acidic phosphorus-based extractant, has significantly different hydrophilic/hydrophobic properties and molecular size compared to the neutral phosphorus-based extractant TBP. To date, no reports on the adsorptive removal of P204 dissolved in water have been published.

To develop an efficient method for removing dissolved P204 from water, we investigated the adsorption and removal performances of two types of ACs for P204 dissolved in water. We also characterized the physicochemical properties of the two ACs. The removal performances of the two ACs for P204 dissolved in water were compared with the performances of two conventional adsorption resins: XAD-4, a nonionic, crosslinked polystyrene resin [37] and IRA900, a macroporous anion-exchange resin [38]. The results provide an important reference for the treatment of wastewater containing P204 in the future.

## 2. Materials and Methods

### 2.1. Materials and Reagents

Coconut shell AC (CSAC; 30–60 mesh, 1000 iodine value) and coal-based AC (CBAC; 30–60 mesh, 1100 iodine value) were purchased from Carbonol Catalytic Technology Co., Ltd. (Pingdingshan, China). Both ACs were washed with water several times and dried before use. IRA900 resin, XAD-4 resin, P204 (AR, 98%), TBP (AR, 98%), sodium chloride (AR, 99.5%), and sodium sulfate (AR, 99%) were purchased from Shanghai Macklin Biochemical Co., Ltd. (Shanghai, China) and used directly. All solutions were prepared with ultrapure water with a resistivity of 18.2 MΩ.

### 2.2. Material Characterization

Fourier-transform infrared (FT-IR) spectrometry (IR Tracer 100, Shimadzu, Kyoto, Japan) was used to detect the functional groups in the CSAC and CBAC samples before and after adsorption. KBr (SP) was used to prepare the samples for FT-IR analysis. The porosity and specific surface area were analyzed by the Brunauer–Emmett–Teller (BET) method using a specific surface area and porosity analyzer (ASAP 2460, Micromeritics, Atlanta, GA, USA). The surface morphology and elemental composition of CSAC samples before and after adsorption were evaluated via scanning electron microscopy and energy-dispersive X-ray spectroscopy (SEM-EDS; Tesscan Mira Lms, Brno, Czech).

### 2.3. Batch Experiments

First, the AC was weighted (0.02 ± 0.0002 g) and added to multiple glass vials. The working solution containing P204 was prepared by dissolving a certain amount of P204 in water, of which the pH value was adjusted by nitric acid. Subsequently, 30 mL of working solution was added to the glass vials and mixed with the AC, and then the glass vials were sealed with Teflon gaps. The samples were placed in a thermostatic air shaker, of which the shaking frequency and the temperature were controlled at 120 rpm and 25 °C, respectively. Once reaching the designated time, the solution was separated from the AC with a microporous filter membrane. The concentration of phosphorus (P) in P204 was measured by an inductively coupled plasma emission spectrometer (ICP-AES, Ultima Expert, Horiba, Kyoto, Japan). According to the concentration change before and after adsorption, the removal efficiency *A* (%) and the adsorption amount *Q* (mg/g) were calculated using the following formula:

(1) $$A=(C_{0}-C_{t})/C_{0}\times 100,$$

(2) $$Q=(C_{0}-C_{t})\times V/m$$

where *C*_0_ (mg/L) represents the initial P204 concentrations, *C_t_* (mg/L) is the P204 concentration at time *t* (h), and *V* (mL) and *m* (g) are the solution volume and adsorbent mass, respectively.

To explore the dominant adsorption mechanism, the data from the batch experiment were further fitted with the pseudo-first order kinetic model (PFO), the pseudo-second order kinetic model (PSO), the Langmuir model, the Freundlich model, or the R-P model. An introduction to these typical mathematical models is described in references [38,39,40].

### 2.4. Column Experiments

**Figure 1:**
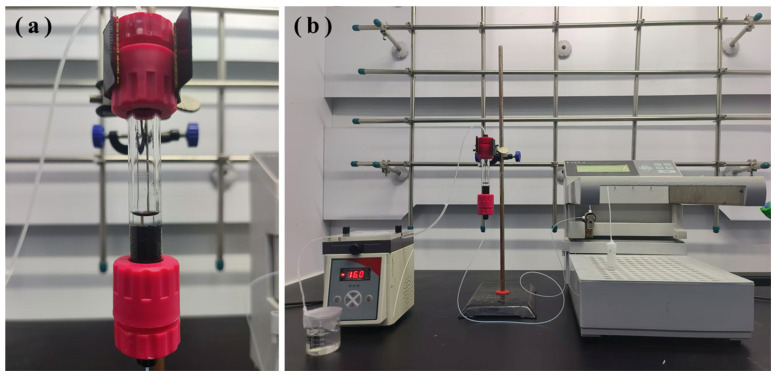
Photos of the column (**a**) and the experimental system (**b**).

For dynamic adsorption, 2.5 mL of CSAC was added to a glass column (dimensions: *h* × *ϕ* = 10 cm × 1 cm) with filter membranes at both ends. The resin column was rinsed with ultrapure water to remove any air bubbles in the column. The liquid level was maintained at approximately 1 cm higher than the resin height. The flow rate was adjusted to 0.50 ± 0.10 mL/min. Subsequently, the working solution containing 19.1 mg/L P ([P204] ≈ 200 mg/L) and 0.1 M HNO_3_ was pumped from the top to the bottom of the column using a peristaltic pump (HL-2B, Jingqi, Shanghai, China). The effluent was collected using an automatic fractionator collector (DC-1500C, EYELA, Tokyo, Japan). Every 10 mL of the effluent was collected in a centrifuge tube for ICP-AES analysis to determine the concentration of P204 at different times. Photos of the column and the experimental system are shown in Figure 1.

For dynamic desorption, 300 mL of working solution containing 18.4 mg/L P ([P204] ≈ 190 mg/L) and 0.1 M HNO_3_ was pumped into the column, followed by 10 mL of 0.1 M HNO_3_ as a rinsing agent. Next, acetone was fed into the column until the fixed P204 was desorbed completely. Other experimental setups were the same as those used in the dynamic adsorption experiment.

## 3. Results

### 3.1. Characterization

**Figure 2:**
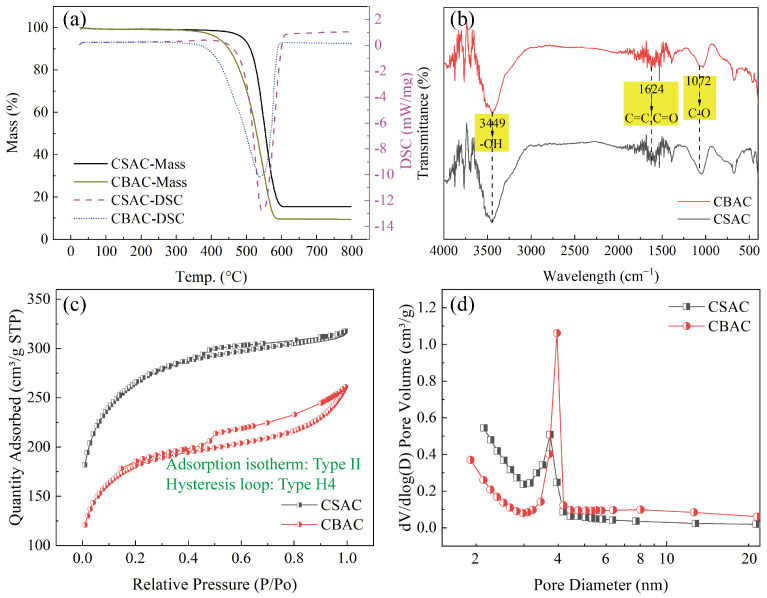
Characterization of the two ACs: (**a**) TG-DSC curves; (**b**) FT-IR spectra; (**c**) N_2_ adsorption and desorption isotherms; and (**d**) pore size distributions.

To fully understand the properties of the two ACs, we first applied thermogravimetric analysis–differential scanning calorimetry (TG-DSC) to evaluate the stability and impurity content of the ACs in an oxygen atmosphere. As shown in Figure 2a, the reaction temperatures of CSAC and CBAC were similar (~550 °C). Before 800 °C, both ACs showed only one exothermic peak corresponding to the chemical reaction between carbon and oxygen, demonstrating that the ACs did not contain other organic substances. However, both CSAC and CBAC showed residual masses after high-temperature calcination, suggesting the presence of inorganic substances in addition to the carbon matrix. The residual mass of CSAC after high-temperature calcination was higher than that of CBAC, indicating a higher content of inorganic components in CSAC.

As shown in Figure 2b, the FT-IR spectra of CBAC and CSAC were essentially the same. The peaks at 3449 cm^−1^ resulted from the stretching vibration of the hydroxyl group in water [41]. The peaks at 1624 cm^−1^ can be attributed to the symmetrical stretching vibration of the C=C group of pyrone and the carboxyl C=O groups [42]. The strong peak at 1072 cm^−1^ is the characteristic peak of C–O [42]. These results indicate that in addition to the carbon matrix, the two ACs contained some oxygen-containing functional groups, consistently with the existing literature [24,43].

Next, we measured the nitrogen adsorption and desorption isotherms of the two ACs [Figure 2c]. CSAC and CBAC both presented Type II nitrogen adsorption isotherms and Type H4 hysteresis loops, indicating large numbers of microporous and non-porous structures. However, the adsorption capacity of nitrogen by CSAC was significantly higher than that of CBAC under the same pressure, suggesting that CSAC had a larger specific surface area. Furthermore, CBAC showed a larger hysteresis loop than CSAC, indicating a higher proportion of microporous structures in CBAC.

According to the measured nitrogen adsorption/desorption isotherms, the pore size distribution was obtained by analyzing the desorption data using the Barrett–Joyner–Halenda model [Figure 2d]. Both ACs exhibited a large number of microporous structures, and the number of micropores increased continuously as the pore size decreased. The number of mesopores was the highest around a diameter of 4 nm, whereas the numbers of mesopores and macropores with diameters exceeding 5 nm could basically be ignored. The specific surface area and pore information of the two ACs are shown in Table 1. The BET specific surface area of CSAC (957 m^2^/g) was significantly larger than that of CBAC (658 m^2^/g). The pore volume of CSAC was larger than that of CBAC, but the average pore diameter of CSAC was slightly smaller than that of CBAC. These results are consistent with those of the nitrogen adsorption/desorption isotherm analysis for the two ACs.

### 3.2. Batch Adsorption Behavior

**Figure 3:**
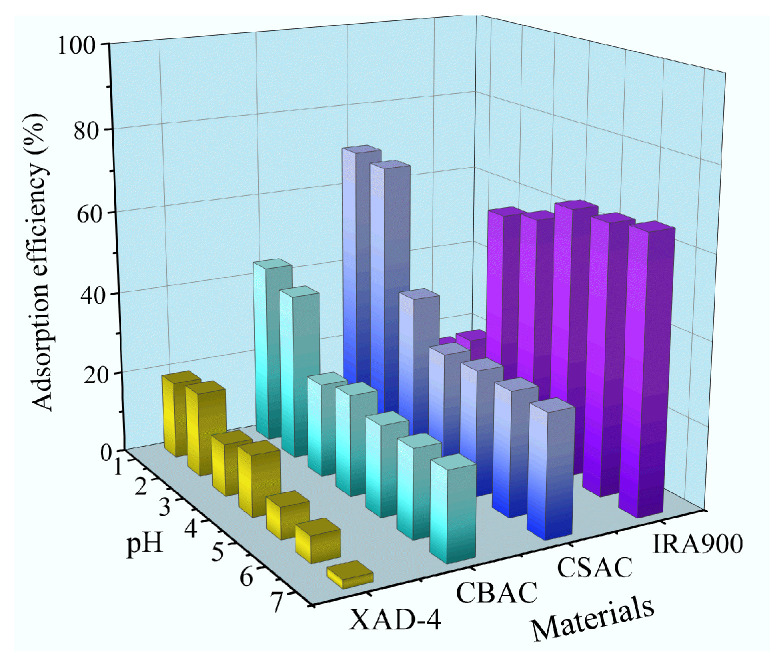
Effects of pH on the P204 adsorption efficiencies of four types of adsorbent materials (*S*/*L* = 0.02 g/30 mL, *t* = 12 h, *T* = 25 °C, [P204] ≈ 200 mg/L).

The adsorption behaviors of the two ACs for P204 dissolved in water were first evaluated under different solution pH values. As shown in Figure 3, the adsorption efficiencies of CSAC and CBAC for P204 dissolved in water both showed downward trends with increasing pH, and they exhibited the best adsorption performance when the pH value was below 1. Considering that the hydrolysis of P204 molecules intensifies with increasing pH, we can infer that both CSAC and CBAC adsorb P204 in the neutral molecular form. CSAC showed better adsorption performance than CBAC; under the same conditions, the removal efficiency of CSAC for P204 dissolved in water was approximately 1.6 times that of CBAC at pH 1. This might be because CSAC had a larger specific surface area and a more suitable pore size, as shown in Table 1. A larger specific surface area means more active sites exposed, and a smaller pore diameter leads to stronger intermolecular forces. The porous adsorption resin XAD-4 showed a similar adsorption behavior to the ACs, but its adsorption performance was inferior. In contrast, the adsorption efficiency of IRA900 resin for water-soluble P204 increased with increasing pH because P204 was deprotonated into an anionic state with increasing pH, making it more readily adsorbed by the anion-exchange IRA900 resin.

**Figure 4:**
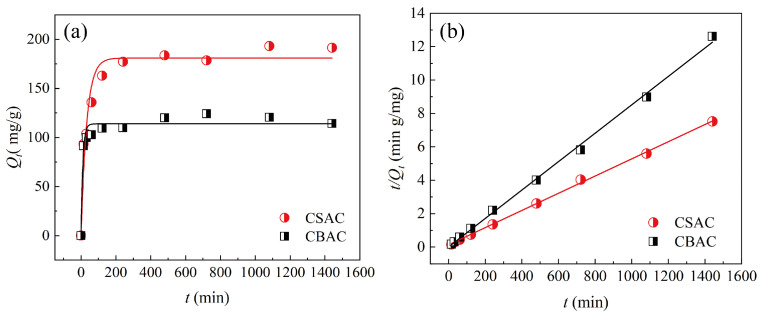
Adsorption kinetics of the two ACs fitted using the (**a**) PFO and (**b**) PSO models (*S/L* = 0.02 g/30 mL, *T* = 25 °C, pH = 1, [P204] ≈ 200 mg/L).

Next, we investigated the adsorption kinetics of the two ACs for P204 dissolved in water. As shown in Figure 4, for both ACs, adsorption equilibrium was reached after approximately 480 min. Before 15 min, the adsorption rates of P204 by both ACs were relatively high, which manifested as a sharp increase in adsorption amount over time. At this stage, the adsorption reaction mainly occurred on the outer surface and in the internal macropores of the adsorbents. Within the range of 15–120 min, the adsorption rate gradually decreased over time. During this stage, the adsorbate gradually diffused into the interior pores of the ACs, and the adsorption reaction occurred within these pores. This process is usually considered to be controlled by the diffusion rate of the solute. After 120 min, the AC adsorption systems gradually approached equilibrium, as demonstrated by the small change in adsorption amount over time. To further explore the adsorption mechanism, the kinetic data of the two ACs were fitted using the PFO and PSO models. The fitting results are shown in Figure 4a,b, and Table 2. According to the fitting parameters (*R*^2^) in Table 2, the adsorption of P204 by both ACs was best described by the PSO model, indicative of chemical adsorption [41].

**Figure 5:**
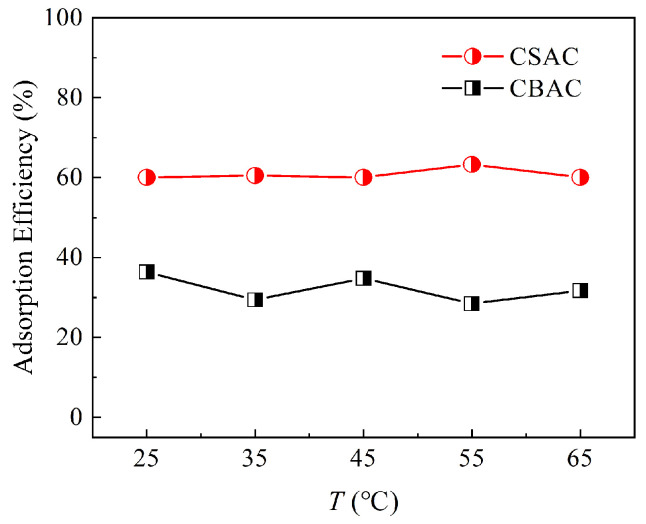
Effects of ambient temperature on P204 adsorption efficiency by the two ACs (*S/L* = 0.02 g/30 mL, pH = 1, *t* = 9 h, [P204] ≈ 180 mg/L).

Figure 5 shows the variation in the removal efficiency of P204 dissolved in water by the two ACs with temperature. Overall, temperature had a minimal effect on the adsorption efficiency of P204 by both ACs. Thus, the tested ACs can be applied in the treatment of wastewater containing P204 within a wide temperature range. Furthermore, these results also suggest that P204 desorption cannot be achieved by increasing or decreasing the temperature.

**Figure 6:**
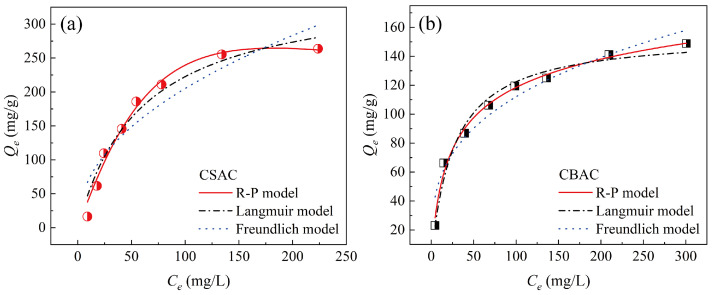
Adsorption isotherms of (**a**) CSAC and (**b**) CBAC (*S/L* = 0.02 g/30 mL, pH = 1, *t* = 9 h, *T* = 25 °C).

Next, we studied the adsorption isotherms of P204 dissolved in water by the two ACs under different initial concentrations of P204. As shown in Figure 6, the adsorption isotherms of P204 by both ACs presented typical concave shapes. Specifically, as the equilibrium concentration increased, the adsorption capacity for P204 increased for both ACs, and adsorption saturation was gradually reached. The experimental maximum adsorption capacity of CSAC for P204 reached 263 mg/g, while that of CBAC was 148 mg/g. To further explore the adsorption mechanism, the Langmuir, Freundlich, and Redlich–Peterson (R–P) models were used to fit the adsorption isotherms. The fitting parameters are listed in Table 3. Based on the correlation coefficients obtained through fitting, the R–P model with three parameters provided the best fit. However, the R-P model has no specific physicochemical significance. Thus, we compared the fitting parameters of the Langmuir and Freundlich models and found that the Langmuir model better described the adsorption process. This suggests that the adsorption of P204 on both ACs occurred via monolayer adsorption [44].

**Figure 7:**
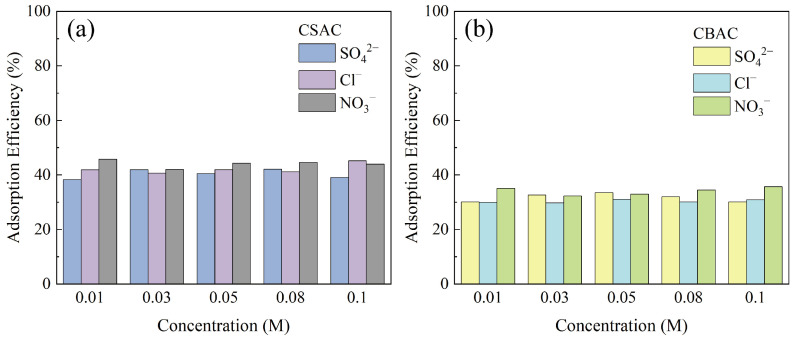
Effects of interfering ions on the P204 adsorption efficiencies of CSAC (**a**) and CBAC (**b**) (*S/L* = 0.02 g/30 mL, pH = 1, *t* = 9 h, *T* = 25 °C, [P204] ≈ 240 mg/L).

Organic wastewater produced by liquid–liquid extraction usually contains coexisting ions. Accordingly, we studied the effects of the types and concentrations of coexisting ions on the adsorption efficiencies of CSAC and CBAC for P204 (Figure 7). For common anions and salts, the removal efficiency of P204 by the two ACs did not change significantly as the ion concentrations increased. This demonstrates that CSAC and CBAC show strong anti-interference ability during the removal of P204.

Overall, the above results indicate that the two ACs have similar adsorption behaviors for water-soluble P204 and exhibit large adsorption capacities, relatively fast adsorption rates, and good performance under both environmental temperature changes and coexisting ions. Compared with CBAC, CSAC showed better removal performance for P204 dissolved in water. Therefore, CSAC was used in the subsequent column adsorption experiments.

### 3.3. Column Experiments

**Figure 8:**
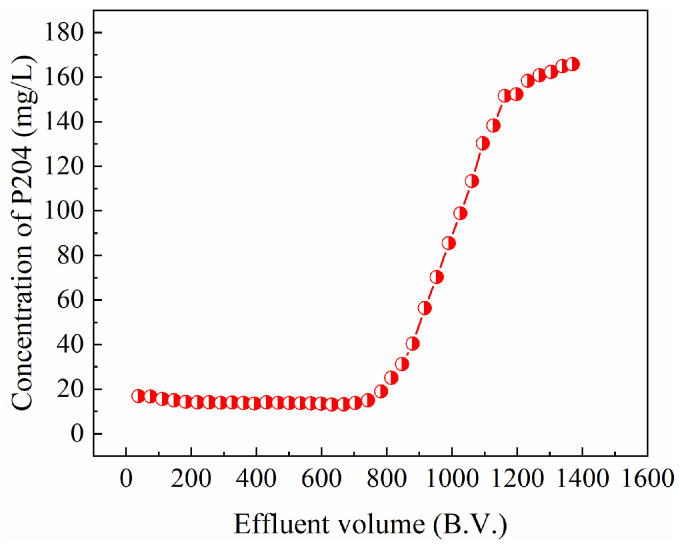
Breakthrough curve of CSAC (column dimensions: *h* × *ϕ* = 10 cm × 1 cm, *V*_CSAC_ = 2.6 mL, *ν* = 0.5 mL/min, [P204] ≈ 200 mg/L, pH = 1).

Dynamic removal performance is an important consideration when evaluating whether a material can be applied in real-world wastewater treatment. The dynamic removal performance of CSAC for water-soluble P204 was evaluated using a glass column filled with CSAC (Figure 8). The dynamic breakthrough curve of P204 in the CSAC-packed column presents a typical S-shaped pattern. In the initial stage, P204 was effectively fixed by the CSAC column, and the concentration of P204 in the effluent dropped below 20 mg/L and then remained constant. When the volume of treated water reached approximately 800 bed volumes (B.V.), the CSAC column began to show penetration, which manifested as the continuous increase in the effluent P204 concentration. When the treatment of water volume reached approximately 1370 bed volumes, the CSAC column was basically saturated.

**Figure 9:**
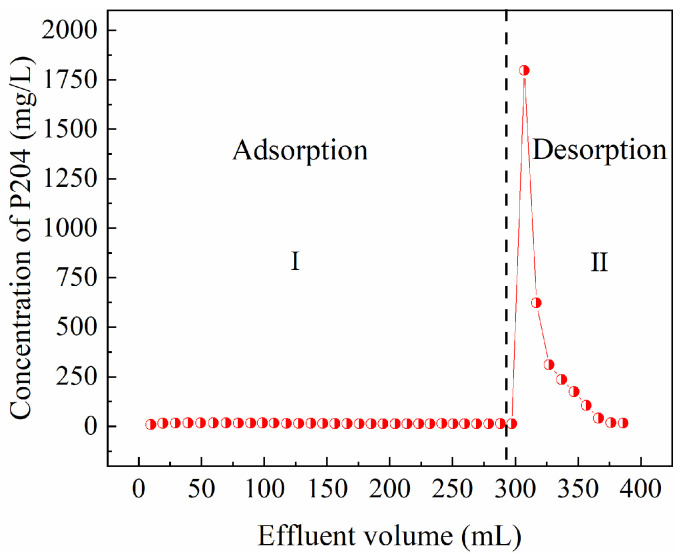
Desorption curve of CSAC (column dimensions: *h* × *ϕ* = 10 cm × 1 cm, *V*_CSAC_ = 2.6 mL, *ν* = 0.5 mL/min, [P204] ≈ 190 mg/L, pH = 1).

To regenerate the spent CSAC and recover the adsorbed P204, we studied the desorption of the CSAC column using acetone. As shown in Figure 9, after acetone was introduced into the resin column, a high-concentration elution peak of P204 immediately appeared in the effluent. Subsequently, the concentration of P204 gradually approached 0. This demonstrates that the P204 fixed on the CSAC adsorption column was successfully desorbed, and the desorbed P204 could be separated from acetone through fractionation to complete the separate recovery of the two organic substances.

**Figure 10:**
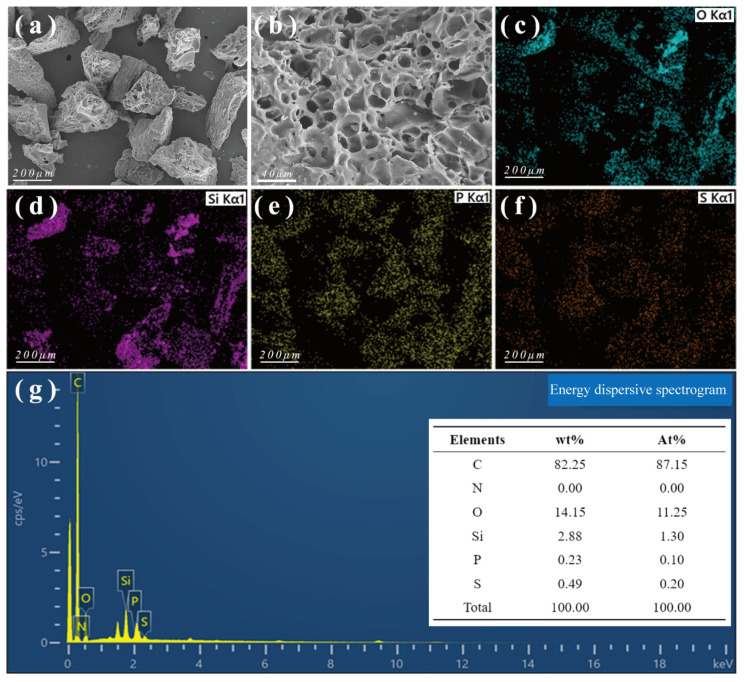
SEM-EDS results for CSAC before adsorption: (**a**,**b**) SEM images; (**c**–**f**) EDS maps showing the elemental distributions; and (**g**) EDS elemental composition data.

CSAC was analyzed by SEM-EDS before adsorption (Figure 10). CSAC exhibited a porous microstructure along with many irregular macropores on the surface, consistent with the BET results. In addition to the carbon matrix, oxygen (O) and silicon (Si) were also found on the surface of CSAC. The presence of O can be attributed to the O-containing functional groups identified by FT-IR spectroscopy. The EDS results also indicated small amounts (<0.2% at) of phosphorus (P) and sulfur (S) in CSAC. The presence of these elements might be due to trace amounts of P and S impurities in CSAC itself or testing errors.

**Figure 11:**
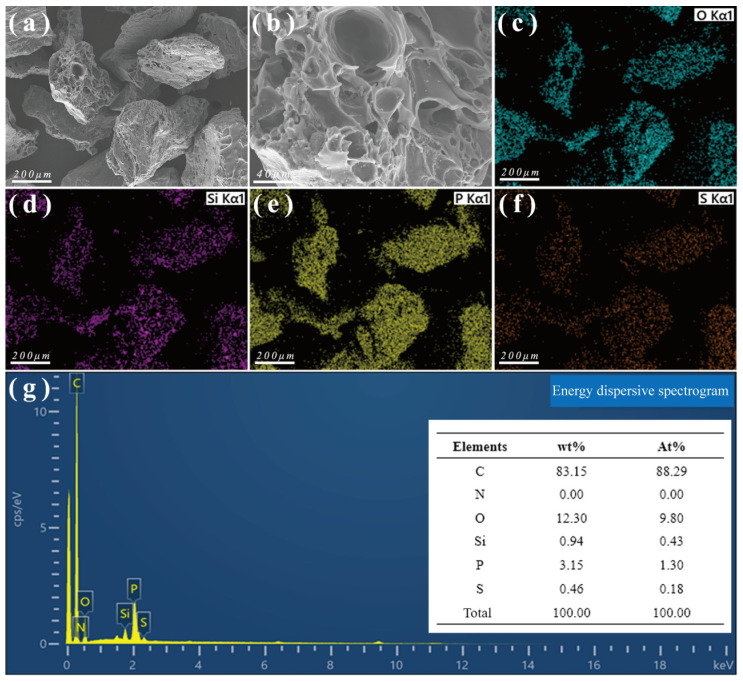
SEM-EDS results for CSAC after adsorption. (**a**,**b**) SEM images; (**c**–**f**) EDS maps showing the elemental distributions; and (**g**) EDS elemental composition data.

Figure 11 shows the SEM-EDS results for CSAC after adsorbing P204. Compared to before adsorption, the content of P in CSAC was significantly higher after adsorption, confirming that P204 was successfully captured by CSAC.

## 4. Conclusions

To address the problem of removing P204 dissolved in water, we characterized two types of ACs (CSAC and CBAC) by TG-DSC, FT-IR spectroscopy, and BET surface area analysis, and then evaluated the P204 removal behavior and performance of the ACs through batch and column experiments. The results suggested that in addition to the main carbon matrix, the two ACs carried rich O-containing functional groups and a small amount of inorganic substances that are not easily oxidized or decomposed (including Si compounds). Both types of AC presented porous structures, with the pores mainly composed of mesopores and micropores with pore diameters less than 4 nm. Both ACs showed Type II nitrogen adsorption isotherms and Type H4 hysteresis loops. The batch experiments showed good removal capabilities of both ACs for P204 under low pH conditions; under the same conditions, the removal efficiencies of CSAC and CBAC were significantly higher than those of the porous adsorption resin XAD-4 and the commercial anion-exchange resin IRA900. The adsorption kinetics of P204 by CSAC and CBAC conformed to the pseudo-second-order model, and the adsorption isotherms conformed to the Langmuir model. Thus, P204 was adsorbed on the two ACs by monolayer chemical adsorption. Both ACs showed strong anti-interference capabilities; their removal performance for P204 did not change significantly with ambient temperature or the concentration of common interfering ions. Under the same experimental conditions, the removal efficiency of P204 was significantly higher for CSAC than for CBAC, potentially because CSAC had a larger specific surface area and a more appropriate pore size range. Column experiments demonstrated that CSAC effectively fixed P204 dissolved in water under dynamic conditions, with a removal efficiency exceeding 90%. Moreover, the fixed P204 could be desorbed with acetone. This paper represents the first systematic exploration of the adsorption behavior and performance of AC for P204 dissolved in water. The results provide an effective strategy for recovering P204 and regenerating spent AC, which shows promise for practical applications in the future.

## Figures and Tables

**Table 1:** BET specific surface area and pore information of two types of AC.

Materials	BET Specific Surface Area (m^2^/g)	Pore Volume (mL/g)	Average Pore Diameter (nm)
CSAC	957	0.49	3.32
CBAC	658	0.40	4.19

**Table 2:** Parameters obtained by fitting the kinetic data with the PFO and PSO models.

Materials	*Q_e_* (exp) (mg/g)	PFO		PSO			
		*Q_e_* (mg/g)	*k*_1_ (min^−1^)	*R* ^2^	*Q_e_* (mg/g)	*k*^2^ (mg·g^−1^·min^−1^)	*R* ^2^
CSAC	193.2	180.9	0.0306	0.957	194.6	0.0019	0.999
CBAC	124.1	113.7	0.0973	0.964	117.6	9.5 × 10^−4^	0.998

**Table 3:** Parameters obtained by fitting the adsorption isotherm data of two types of AC.

Materials	Langmuir Model			Freundlich Model			R-P Model			
	*Q_m_*	*K_L_*	*R* ^2^	*K_F_*	*n*	*R* ^2^	*K_R_*	*a*	*b*	*R* ^2^
CSAC	354.7	0.0169	0.963	23.98	2.15	0.876	4.27	7.39 × 10^−4^	1.51	0.984
CBAC	155.6	0.0369	0.979	26.54	3.20	0.950	8.98	0.121	0.869	0.992

## Data Availability

The data presented in this study are available on request from the corresponding author.
